# Malignant Odontogenic Tumours: A Systematic Review of Cases Reported in Literature

**DOI:** 10.3389/froh.2021.775707

**Published:** 2021-11-19

**Authors:** Constanza Marin, Manas Dave, Keith D. Hunter

**Affiliations:** ^1^Unit of Oral and Maxillofacial Medicine, Pathology and Surgery, University of Sheffield, Sheffield, United Kingdom; ^2^Unidad de Patología y Medicina Oral, Facultad de Odontología, Universidad Andres Bello, Viña del Mar, Chile; ^3^Division of Dentistry, The University of Manchester, Manchester, United Kingdom; ^4^Department of Oral Pathology and Biology, School of Dentistry, University of Pretoria, Pretoria, South Africa

**Keywords:** odontogenic tumour, malignant odontogenic tumour, radiographic features, treatment, recurrence

## Abstract

**Background:** Malignant odontogenic tumours (MOTs) arise either *de novo* from the tooth forming tissues, their developmental residues or from existing odontogenic epithelial or mesenchymal neoplasms in the jaws. Their management requires extensive surgery due to their infiltrative nature and risk of metastasis. There is a need to understand the clinical and pathological features of MOTs to inform both treatment algorithms and prognostication. This is an area of diagnostic pathology which presents substantial difficulties in diagnosis, compounded by inconsistent use of terminology. Thus, this systematic review aimed to describe the clinical and pathological features of MOTs with a view to consolidating the literature and defining problematic areas in diagnosis and classification.

**Methods:** An electronic database search was conducted in Web of Science, PubMed/Medline, and Embase. Additionally, the grey literature and reference lists of selected papers searched for completeness. Nine hundred and sixty articles were initially identified. Following removal of duplicates and application of inclusion/exclusion criteria, 312 articles were included for qualitative analysis.

**Results:** The 312 articles encompassed a total of 507 patients with most lesions located within the mandible (74.3%). The most common first histological diagnosis was ameloblastic carcinoma (25.7% of all diagnoses), but there is considerable variation in how and when various diagnostic terms are used, and several misdiagnoses were reported. An initial benign diagnosis was made in 24.7% of patients, followed by a later malignant diagnosis and in this sub-group, the most common benign first diagnosis was ameloblastoma (42.4%). Cervical lymph nodes were the most common site of metastasis (9.3% of patients). With respect to distant metastasis (DM), the lungs were the most common organ affected (11.2% of DM patients) with metastasising ameloblastoma the most commonly reported tumour which metastasised to the lungs. Overall, 26.8% of patients developed recurrence.

**Conclusion:** Overall, the quality of the literature on MOTs is poor. This review of the literature has highlighted variations in diagnostic terms and criteria which has resulted in areas of confusion with potential for misdiagnosis. This consolidation of primary data has identified key areas for targeted research including further discussion on the malignant potential of ameloblastoma.

## Introduction

**Figure 1:**
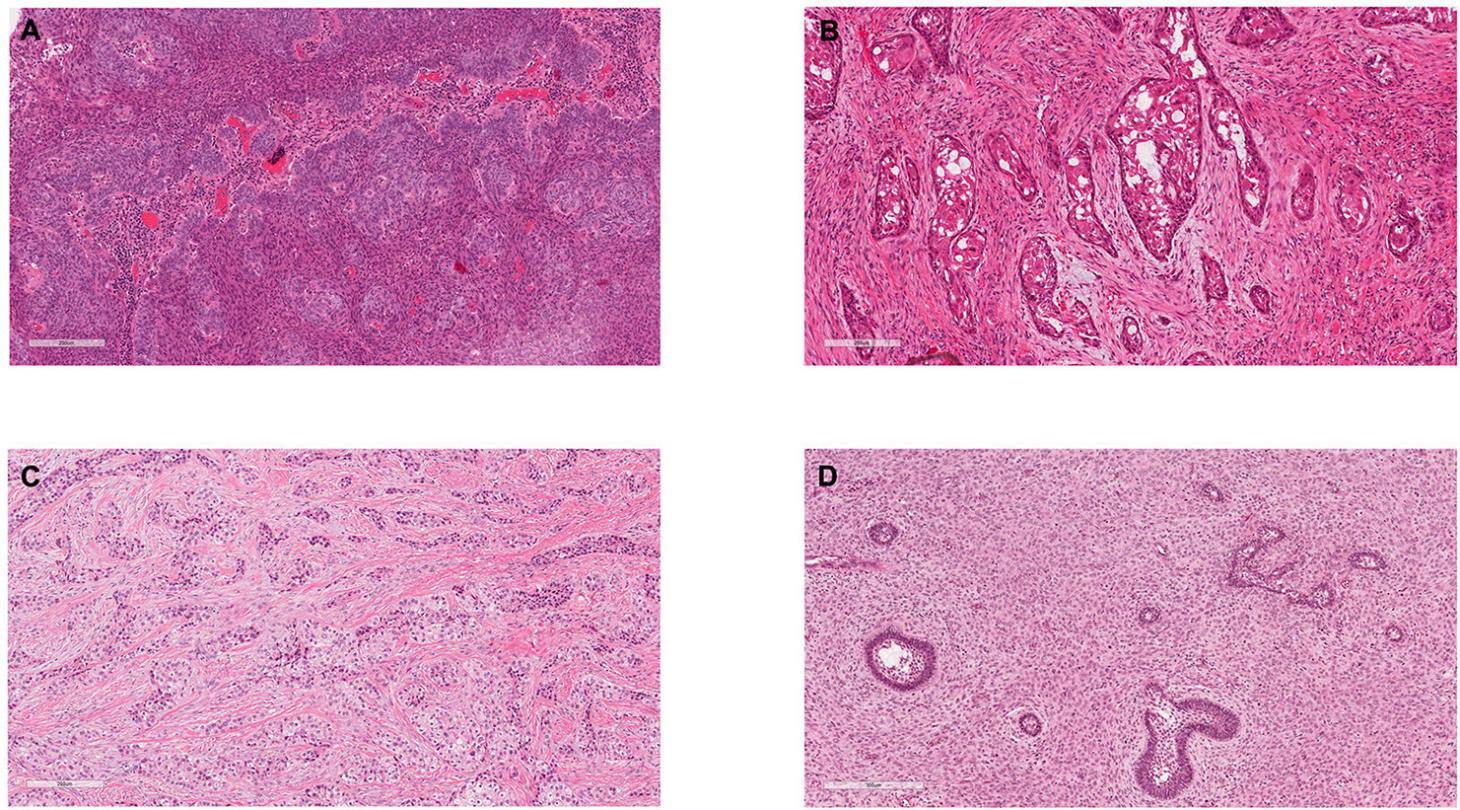
Exemplar histology of the commonest malignant odontogenic tumours. **(A)** Ameloblastic carcinoma. This was a maxillary lesion and the patient presented with a lymph node metastasis within 6 months. **(B)** Primary Intraosseous Carcinoma NOS (historically synonymous with PIOSCC) from the angle of the mandible. **(C)** Clear Cell Odontogenic Carcinoma, which showed re-arrangement of the EWSR1 gene. **(D)** Ameloblastic Fibrosarcoma.

Malignant odontogenic tumours (MOTs) arise either de novo from the tooth forming tissues, their developmental residues or from existing odontogenic epithelial or mesenchymal neoplasms in the jaws and malignant transformation of benign odontogenic tumours (OTs) [1, 2]. Most MOTs are neoplasms consisting of malignant epithelium, including ameloblastic carcinoma and primary intraosseous squamous cell carcinoma (PIOSCC) [2–5] (Figure 1). The frequency of MOTs varies across geographic regions comprising 0.3–0.4% of all OTs in Brazil [6, 7], 1.1–1.3% in Nigeria and Mexico [5, 8] and accounting for 5.7% in the United Kingdom [3]. However, the published case series are very variable, as some are based on referral cases rather than a true representation of population incidence, and odontomes are variably included, which can dramatically affect the overall proportions of individual tumours [3]. A variety of growth patterns have been reported from slow-growing to highly aggressive tumours with local recurrence and metastasis, most commonly to cervical lymph nodes and lungs [2, 9, 10]. Therefore, management may require extensive surgery and adjuvant therapies such as radiotherapy and/or chemotherapy.

MOTs present several challenges in terms of classification, diagnosis and prognosis. The World Health Organisation (WHO) updated their Classification of Head and Neck Tumours in 2017, simplifying the MOT classification and adding the newly described tumour, Sclerosing Odontogenic Carcinoma [11]. Diagnostic criteria for MOTs have developed and changed over several iterations of the WHO classification, but for some entities, these criteria are still rather vague. Furthermore, genetic profiles and biomarkers have also been described for some tumour entities in recent years [11, 12]. The diagnostic challenges of MOTs are due to similar histopathological appearances to their benign counterparts and limitations of incisional biopsies not being representative of the entire tumour [3], leading to misdiagnosis in the first histopathological sample.

Currently, due to the limited number of a wide variety of reported cases and their classification, neither outcome nor prognosis can be confidently outlined. Thus, there is a need to understand the clinical and pathological features of MOTs to inform both treatment algorithms and prognostication. This systematic review aims to determine these features with a view to consolidate and explain the problems in the literature, delineate current problems and inform future research.

## Methods

A preliminary literature search was conducted by one of the authors (MD) to inform the search strategy. Keywords were based on the current and historic classification of tumour names to include studies with differences in nomenclature but those that may still fulfil the inclusion criteria.

An electronic search of the following databases was conducted; Web of Science, PubMed/MEDLINE and Embase from 1946 or 1974 respectively to August 2020 with the following key words: (odontogenic carcinoma) OR (malignant ameloblastoma) OR (metastasizing/metastasising ameloblastoma) OR (ameloblastic carcinoma) OR (sclerosing odontogenic carcinoma) OR (primary intraosseous carcinoma) OR (primary intraosseous squamous cell carcinoma) OR (malignant variants of odontogenic tumours) OR (malignant changes in odontogenic cysts) OR (odontogenic cyst malignancy) OR (clear cell odontogenic carcinoma) OR (ghost cell odontogenic carcinoma) OR (malignant keratocyst) OR (malignant keratocystic odontogenic tumour) OR (odontogenic sarcoma) OR (ameloblastic fibrosarcoma) OR (ameloblastic odonto-dentinosarcoma) OR (ameloblastic odontosarcoma) OR (ameloblastic fibrodentinosarcoma) OR (ameloblastic fibroodontosarcoma) OR (odontogenic carcinosarcoma) OR (malignant changes in odontogenic cysts). Additionally, a search of the grey literature was undertaken through several sources including google scholar, professional pathology society webpages, government guidelines and reports and relevant hospital publications. This was supplemented by screening entire reference lists of included studies. This search strategy conforms to the preferred reporting items for systematic reviews and meta-analysis (PRISMA) and is registered on PROSPERO (CRD42021248757).

Parameters were kept broad to maximise search results. The inclusion criteria consisted of full-text primary research studies on humans which were related to the development of malignancy in odontogenic cysts as confirmed histologically. Only studies in English and Spanish were included as they could be reliably interpreted by the research team. Case reports and case series with small cohorts were permissible given the rarity of MOTs if they extracted the minimum dataset for each patient, including tumour site, histological diagnosis and patient treatment. On the other hand, case series where individual patient parameters could not be defined were not included. Similarly, *in-vitro* studies or MOTs reported in animals were also excluded. Conference abstracts or items where the full-text was unavailable were also excluded.

All extracted data were initially stored and imported into EndNote (Philadelphia, United States of America) and transferred into a Microsoft Office Excel spreadsheet (Washington, United States of America). Screening of studies was undertaken using Covidence (Melbourne, Australia). Due to the heterogeneity between studies, analysis was limited to descriptive statistics. Risk of bias assessment was not deemed necessary given most studies were anticipated to be cohort/case series and with the rarity of MOTs and no universally agreed treatment algorithm, the risk of bias was uncertain on almost all reported cases and quality of evidence low. The data extraction form was adapted from Chrcanovic et al. [13].

The terms malignant ameloblastoma, ameloblastic carcinoma and metastasizing ameloblastoma have been used inconsistently and somewhat interchangeably in literature. For data extraction, we adopted the authors' reported diagnosis and acknowledged ameloblastoma as a malignant entity (which fulfilled our inclusion criteria) if it metastasised or was reported to have cytological atypia, albeit these features, of themselves, are insufficient for a definitive diagnosis of malignancy (this issue will be discussed later). Data repositories, such as the Surveillance, Epidemiology, and End Results (SEER) database use ICD-10 coding of tumours for collation of data. This has led to ambiguity and confusion, as the ICD-10 classification does not map well onto the WHO classification of tumours (neither 2005 or 2017), utilising different terms and grouping entities in a different manner [4, 14, 15]. This topic will be further developed in the discussion.

## Results

**Figure 2:**
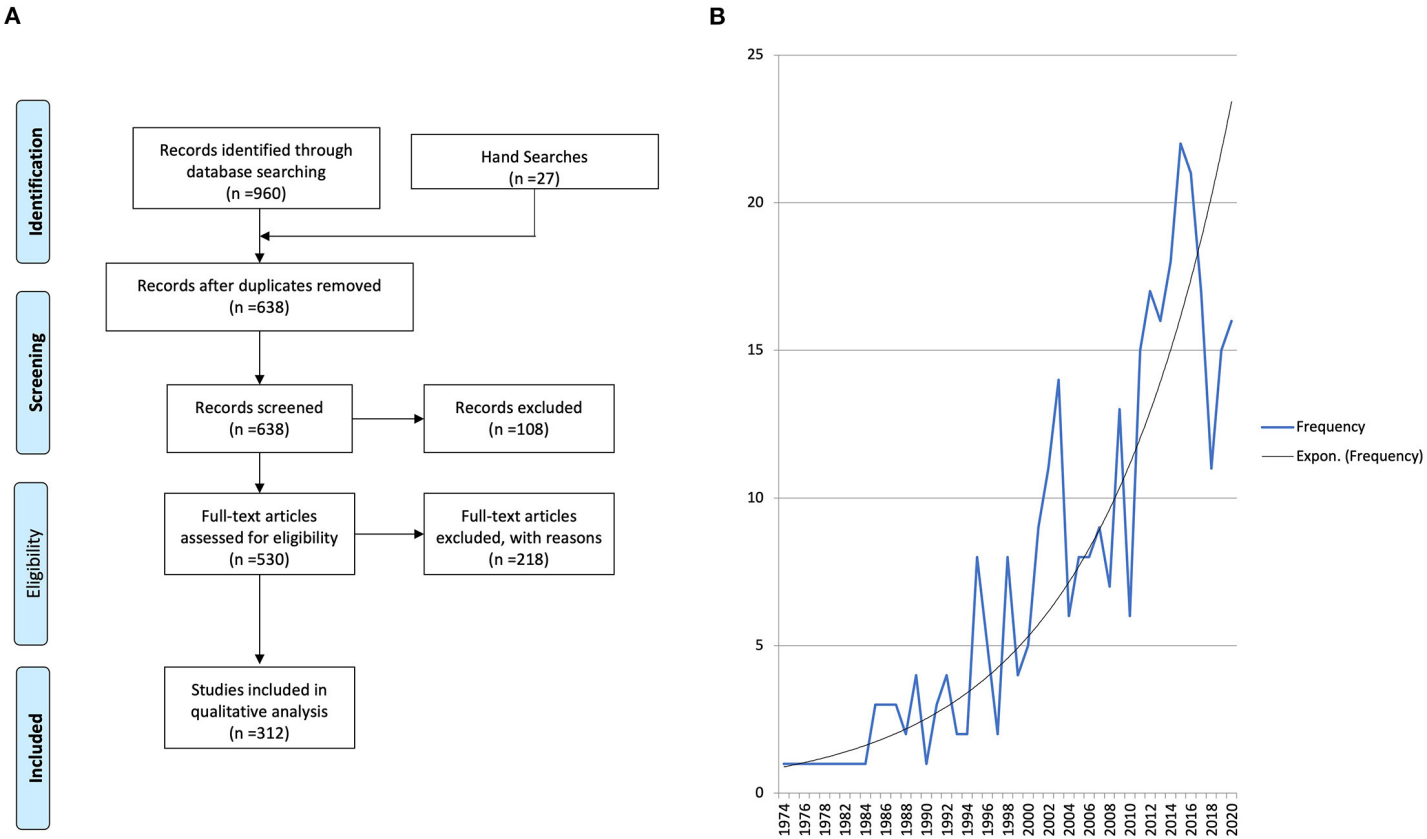
**(A)** PRISMA flow diagram. Two hundred and eighteen articles were excluded with the following reasons: full text not available (*n* = 103), no treatment details (*n* = 29), no primary data (*n* = 20), conference abstract only (*n* = 18), unable to extract individual patient data (*n* = 13), insufficient clinical information (*n* = 12), articles not in English or Spanish (*n* = 9), molecular/immunohistochemistry studies only (*n* = 8), no malignant odontogenic tumour present (*n* = 4), and non-human cases (*n* = 2). **(B)** Frequency of publications included in this review over time with an exponential line of best fit traced.

The search results are outlined in Table 1 and Figure 2A. There were 960 studies identified in the initial literature search and a further 27 studies by manual hand searching. Following duplicate removal (*n* = 349), 638 studies had their titles and abstracts screened by two reviewers (MD and CM) to ensure they satisfied the inclusion and exclusion criteria. If there was any doubt at this stage, the studies were included for full-text review. One hundred and eight articles were excluded as they were not relevant, and 530 articles had a full-text review. Any disagreements regarding inclusion/exclusion were resolved through discussion and the input of a third reviewer (KH) who made the final decision. Two hundred and eighteen articles were excluded with the following reasons: full text not available (*n* = 103), no treatment details (*n* = 29), no primary data (*n* = 20), conference abstract only (*n* = 18), unable to extract individual patient data (*n* = 13), insufficient clinical information (*n* = 12), articles not in English or Spanish (*n* = 9), molecular/immunohistochemistry studies only (*n* = 8), no malignant odontogenic tumour present (*n* = 4) and non-human cases (*n* = 2). In total, 312 articles were included in this systematic review (Supplementary Material).

**Table 1:** Summary of the search strategy.

**ID**	**Key word**	**Search results**
1	Odontogenic carcinoma	365
2	Malignant ameloblastoma	227
3	Metastasising ameloblastoma	2
4	Metastasizing ameloblastoma	40
5	Ameloblastic carcinoma	32
6	Sclerosing odontogenic carcinoma	36
7	Primary intraosseous carcinoma	140
8	Primary intraosseous squamous cell carcinoma	87
9	Malignant variants of odontogenic tumours	0
10	Malignant changes in odontogenic cysts	0
11	Odontogenic cyst malignancy	0
12	Clear cell odontogenic carcinoma	165
13	Ghost cell odontogenic carcinoma	64
14	Malignant keratocyst	0
15	Malignant keratocystic odontogenic tumour	0
16	Odontogenic Sarcoma	30
17	Ameloblastic fibrosarcoma	167
18	Ameloblastic odonto-dentinosarcoma	0
19	Ameloblastic odontosarcoma	16
20	Ameloblastic fibrodentinosarcoma	16
21	Ameloblastic fibroodontosarcoma	3
22	Odontogenic carcinosarcoma	18
23	1 or 2 or 3 or 4 or 5 or 6 or 7 or 8 or 9 or 10 or 11 or 12 or 13 or 14 or 15 or 16 or 17 or 18 or 19 or 20 or 21 or 22	960

The 312 studies included 507 patients with an average age of 45.8 years (range 4 months−90 years) with 63% male and 37% female. More MOTs were located in the mandible (74.3%) compared to the maxilla (25.7%). Tobacco and alcohol intake and previous history of malignancy were variably reported which precluded analysis, as this would not be representative of the included cohort.

The date of publication ranged from 1974 to 2020 with an increase in the number of published MOT case reports and series over time (Figure 2B).

### Symptoms

Symptoms were reported for 146 patients (28.8%). The average duration of symptoms before a malignant diagnosis was 14.8 months (range 2 days−600 months) and included swelling (painful or painless), paraesthesia/dysaesthesia, fever, trismus, oral discharge, failure of healing, facial deformity and tooth mobility.

### Histological Diagnosis

**Figure 3:**
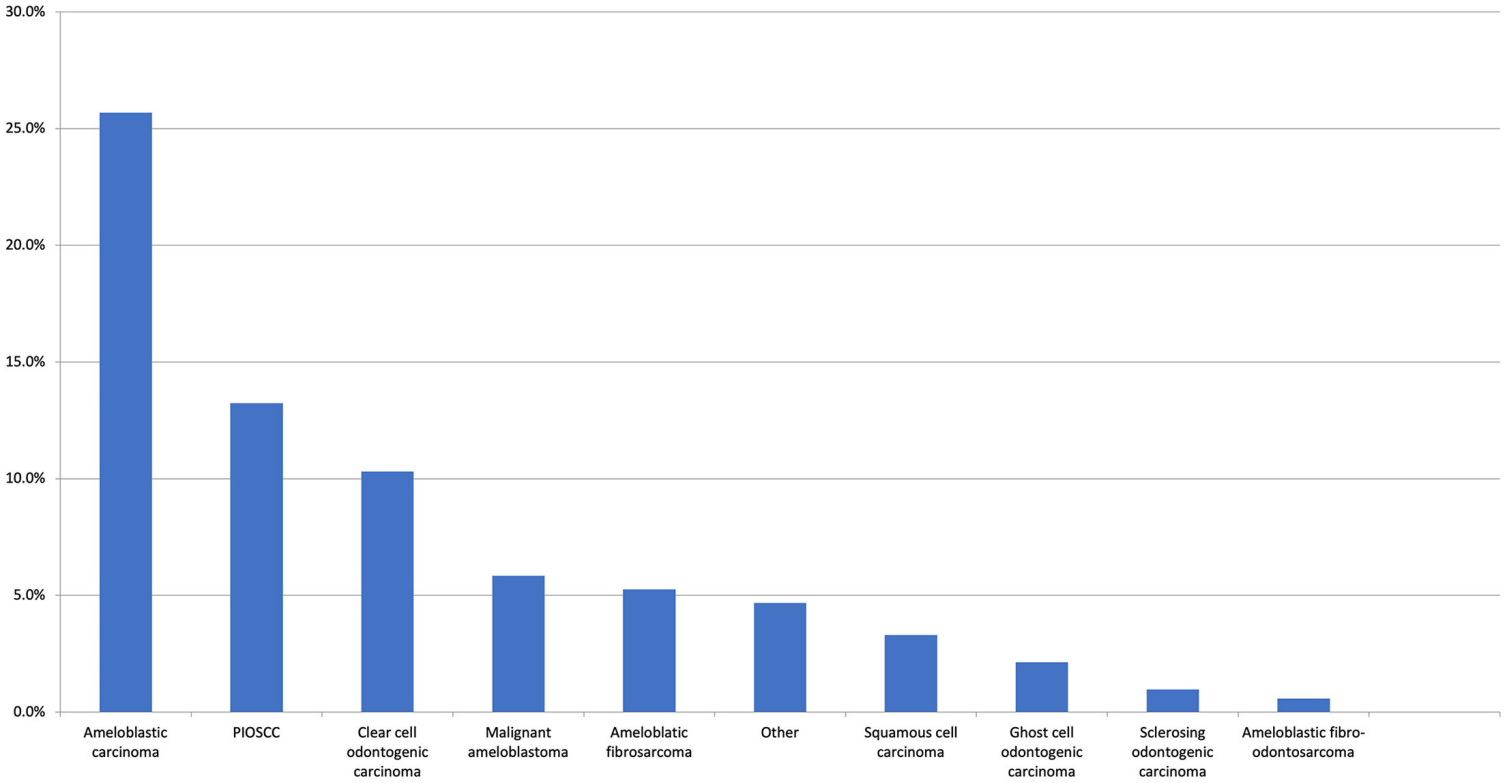
The top 10 most common malignant first histological diagnoses. The “Other” category includes clear cell carcinoma (0.6%), adenocarcinoma NOS, metastatic carcinoma, and undifferentiated (all 0.4%). There were 13 other malignant entities reported at the same frequency (all 0.2%).

**Figure 4:**
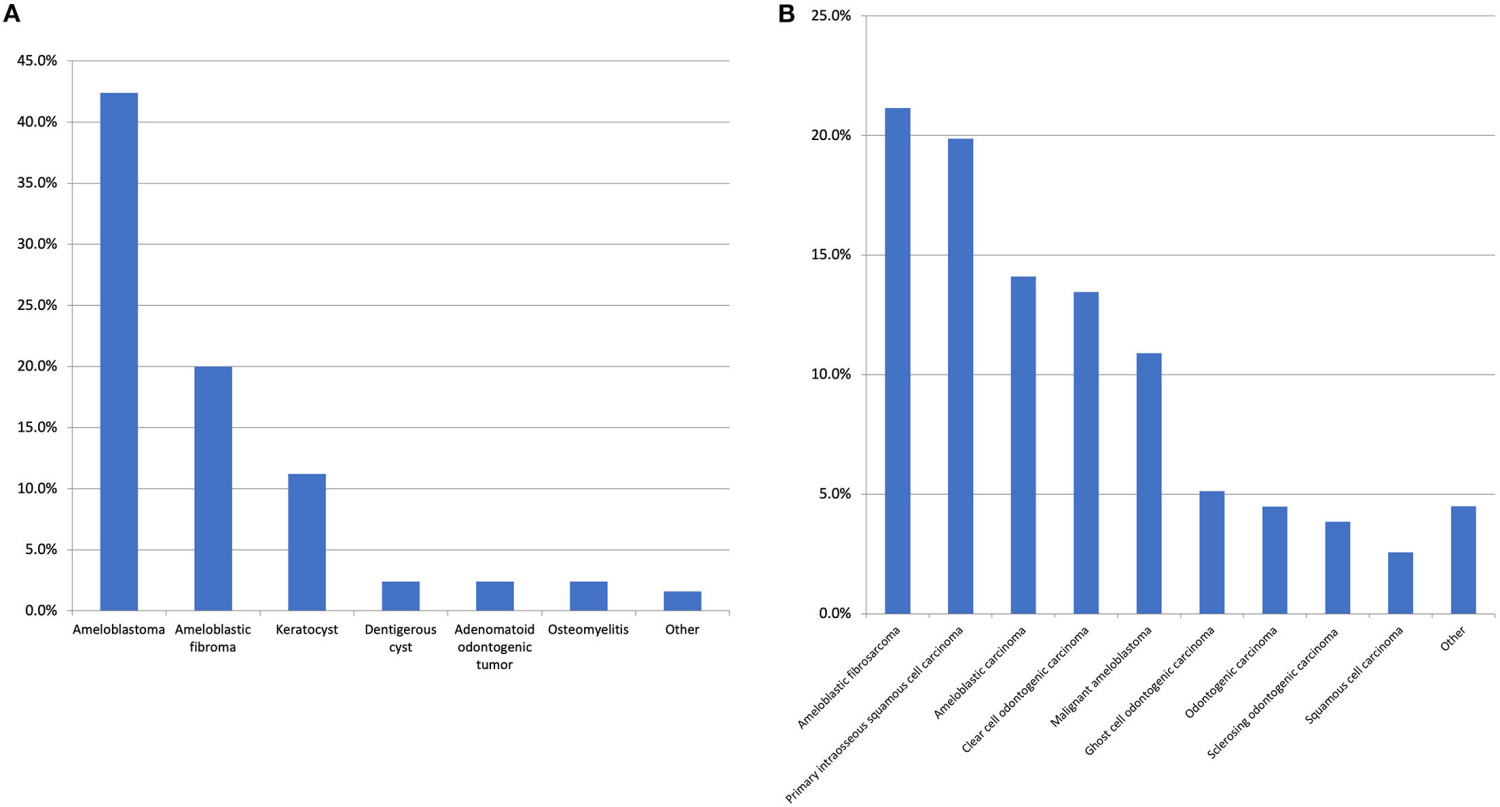
**(A)** The most common benign first histological diagnosis. The “other” category includes unspecified odontogenic cyst, calcifying epithelial odontogenic tumour, cemento-ossifying fibroma, inflammation/infection post tooth-extraction, squamous odontogenic tumour, and pleomorphic adenoma with all with the same frequency (1.6%). There were 17 other diagnoses made with a range of conditions such as unspecified ameloblastic tumour and unspecified benign odontogenic cyst which were all reported at the same frequency (0.8%). **(B)** Top 10 most common second histological diagnosis. The “other” category includes ameloblastic fibro-odonto sarcoma, odontogenic carcinosarcoma (both 1.3%) and ameloblastic dentinosarcoma, malignant Pindborg tumour and calcifying epithelial odontogenic tumour (all 0.6%). It is of note, regarding the case of calcifying epithelial odontogenic tumour, one was diagnosed as a malignant variant and one case was diagnosed as a clear cell odontogenic carcinoma initially however this diagnosis was revised to the aforementioned on the resection.

The preliminary literature search identified numerous published cases which had an initial benign histological diagnosis followed by a subsequent recurrence or malignant transformation. Therefore, the first and second histological diagnoses were captured in our data collection, but this issue significantly complicated the assessment of the literature. If the original diagnosis remained unchanged, no second histological diagnosis was provided. If multiple diagnoses were made, the first and final diagnoses were recorded. In total, 60 unique diagnoses were made with the top 10 most common malignant first histological diagnosis presented in Figure 3 and the most common benign first histological diagnoses (before a subsequent malignant diagnosis) presented in Figure 4A.

With respect to the first histological diagnosis, the most common malignant diagnosis was ameloblastic carcinoma (25.7%), followed by primary intraosseous squamous cell carcinoma (13.2%), clear cell odontogenic carcinoma (10.3%), malignant ameloblastoma (5.8%), and ameloblastic fibrosarcoma (5.3%). An initial benign diagnosis was made for 24.7% of all patients. The most common initial benign diagnosis (expressed as a percentage of all first benign diagnoses) was ameloblastoma (42.4%), ameloblastic fibroma (20%), odontogenic keratocyst (11.2%), adenomatoid odontogenic tumour (2.4%), dentigerous cyst (2.4%), and osteomyelitis (2.4%). From this initial benign diagnosis, the most common malignant neoplasms in the second histological diagnosis were ameloblastic fibrosarcoma (25.6%), primary intraosseous squamous cell carcinoma (20.5%), malignant ameloblastoma (14.5%), ameloblastic carcinoma (14.5%), and clear cell odontogenic carcinoma (9.4%). With respect to the second definitive histological diagnosis, there were 14 unique diagnoses made, with the top 10 presented in Figure 4B.

**Figure 5:**
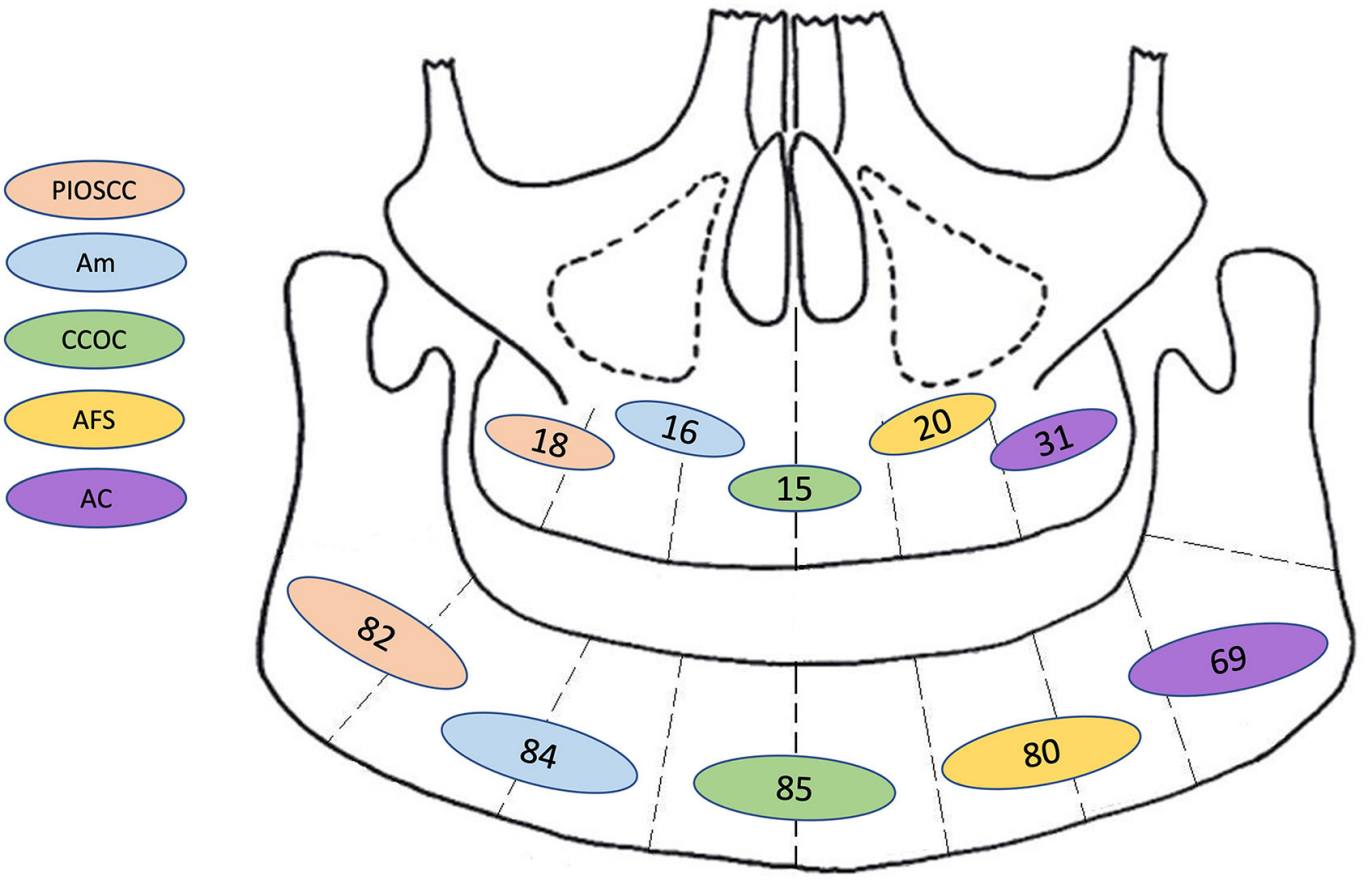
Graphic representation for frequency of site distribution (by jaw only: mandible or maxilla) of the most common combined first and second malignant histological diagnosis. PIOSCC, primary intraosseous squamous cell carcinoma; Am, malignant ameloblastoma; CCOC, clear cell odontogenic carcinoma; AFS, ameloblastic fibrosarcoma; AC, ameloblastic carcinoma; N.B, This diagram only illustrates tumour frequencies in the mandible and maxilla, further information for sub site/laterality was not available.

With both the first and second histological diagnoses combined, the most common diagnoses were ameloblastic carcinoma (23.0%), primary intraosseous squamous cell carcinoma (14.8%), clear cell odontogenic carcinoma (11.0%), ameloblastic fibrosarcoma (9.0%) benign ameloblastoma (7.9%), and malignant ameloblastoma (7.0%). It is of note that whilst ameloblastomas are considered benign entities, the aforementioned “malignant ameloblastomas” either metastasised or were reported as malignant based on the degree of cytological atypia. The latter cases were not called ameloblastic carcinoma by the authors but described as ameloblastoma with high grade morphology or with atypia inferring malignant phenotypic changes. It is not clear in many cases why the term ameloblastic carcinoma was not used, nonetheless cases describing a “malignant” ameloblastoma were included as meeting the criteria of a malignant odontogenic tumour. This is not a homogeneous group, as the term has been variably applied, but addressing this issue would have required detailed re-analysis/diagnosis of every published case. To ensure clarity in data extraction, the author's diagnosis was recorded as stated. Further sub-group analysis was undertaken of these top five most common malignant diagnoses summarised in Table 2. This was adjusted with respect to the final recorded histological diagnosis to prevent overlapping of data. A graphic representation for frequency site distribution of MOTs is presented in Figure 5.

**Table 2:** Summary of subgroup analysis for the combined first and second histological diagnoses.

	**PIOSCC/IOC(NOS)**	**"Malignant” ameloblastoma**	**Clear cell odontogenic carcinoma**	**Ameloblastic fibrosarcoma**	**Ameloblastic carcinoma**
Mean age (years, with range)	53.1 (6–84)	38.6 (8–75)	55.9 (25–89)	27.3 (0.3–83)	45.6 (2–90)
Sex ratio (M:F)	67:28	11:6	14:23	35:23	33:16
Location	82.3% mandible 17.7% maxilla	84.3% mandible15.7% maxilla	85.3% mandible 14.7% maxilla	79.7% mandible20.3% maxilla	68.5% mandible 31.5% maxilla
Malignant diagnosis sample	Biopsy 35.4% Resection 64.6%	Biopsy 37.0%Resection 63.0%	Biopsy 48.7% Resection 51.3%	Biopsy 31.6%Resection 68.4%	Biopsy 63.3% Resection 36.7%
Surgery (neck dissection)	98.9% (54.0%)	98.0% (16.3%)	94.4% (29.2%)	98.2% (5.3%)	97.1% (21.0%)
Chemotherapy	14.9%	12.2%	6.9%	17.5%	5.1%
Radiotherapy	37.9%	14.3%	23.6%	24.6%	26.1%
Chemotherapy and radiotherapy	5.7%	2.0%	0.0%	1.8%	1.4%
Surgery and chemotherapy	13.8%	12.2%	2.8%	15.8%	3.6%
Surgery and radiotherapy	37.9%	14.3%	23.6%	24.6%	25.4%

*The diagnosis was only included in the analysis if it was the final diagnosis stated by the authors. Patients who declined treatment were removed from final treatment analysis*.

### Metastasis Site

In total, 36.7% of all patients had reported metastasis. Cervical lymph nodes were the most common site of metastasis with 9.3% of patients having positive nodes on first presentation. A further 3.2% of patients developed cervical lymph node metastasis as their disease progressed.

The following MOTs were reported with respect to lymph node metastases (percentage expressed as a proportion of all cases with lymph node metastases only); PIOSCC (33.8%), ameloblastic carcinoma (21.6%), malignant ameloblastoma (16.2%), clear cell odontogenic carcinoma (14.9%), squamous cell carcinoma (4.1%), and ghost cell odontogenic carcinoma and ameloblastic fibrosarcoma (both 2.7%), and spindle cell carcinoma, ameloblastic fibrosarcoma and malignant calcifying epithelial odontogenic tumour (all 1.4%).

**Figure 6:**
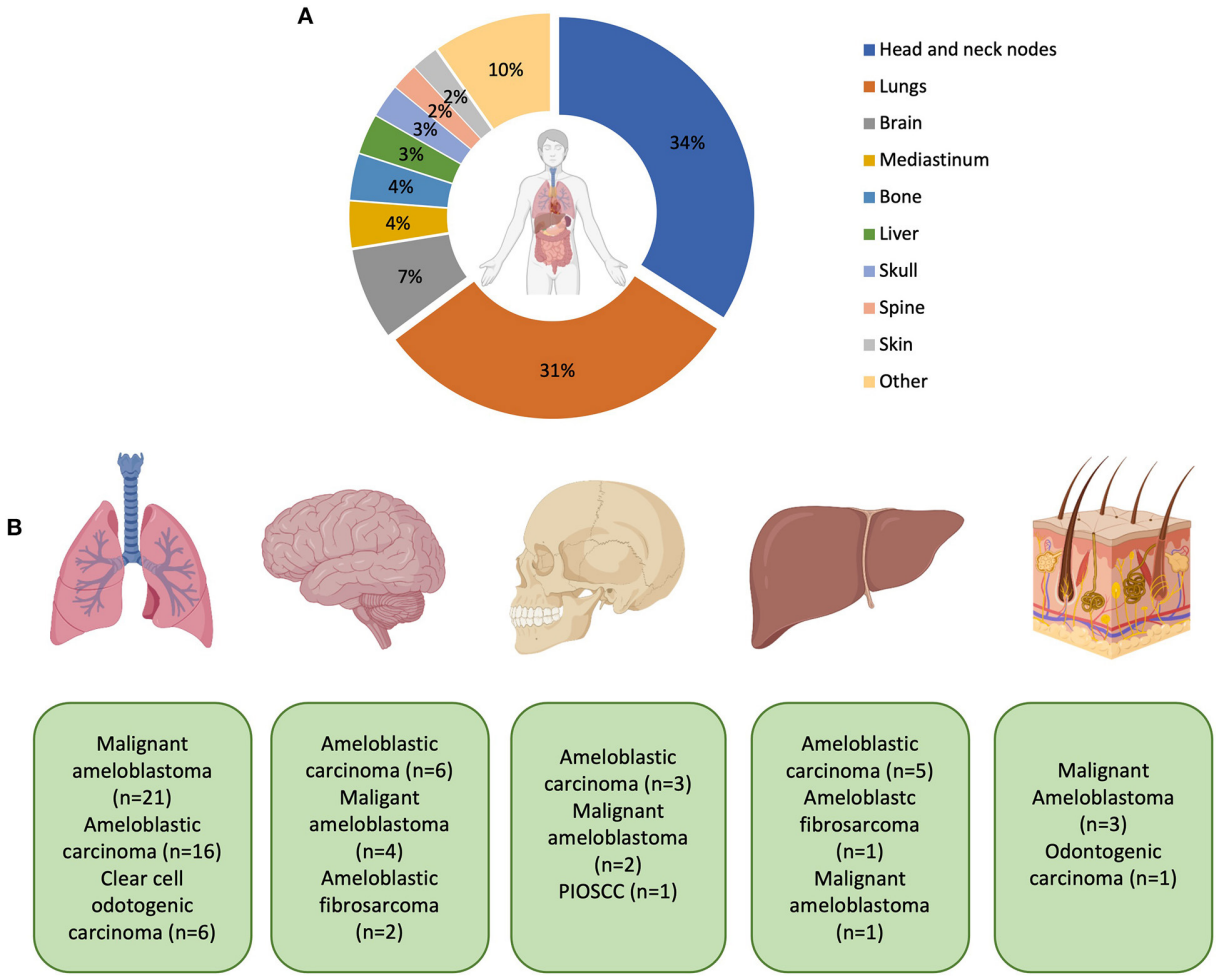
**(A)** Representation of the most common sites of metastasis. Percentages are expressed to only reflect patients who have had metastases. Patients with no recorded metastasis are not included in this calculation. The “other” category includes the thyroid, kidneys, non-cervical lymph nodes, and cranial nerves (all 1.1%) and salivary glands, pharynx, orbit, larynx, intestines, heart, branchial plexus, carotid bifurcation, and unspecified distant metastasis (all 0.5% respectively). **(B)** Graphic representation of the most common MOTs metastasising to distant sites (from left to right: lungs, brain, skull, liver and skin–mediastinum was not included in the diagram). With metastasis to the lung, ameloblastic fibrosarcoma (*n* = 2), squamous cell carcinoma (*n* = 2), unspecified odontogenic biphasic malignant neoplasm (*n* = 1), and unspecified odontogenic carcinoma (*n* = 1) were also recorded but not shown in the diagram. With metastasis to the brain, clear cell odontogenic carcinoma and unspecified odontogenic carcinoma were both recorded (both *n* = 1) but also not shown in the diagram. Created with BioRender.com.

The lungs were the most common organ to be affected by distant metastasis affecting 11.2% of all patients (Figure 6). Subgroup analysis of patients with lung metastasis determined that the most common histological diagnosis (including 1st and 2nd histological diagnoses; adjusted by removing the first histological diagnosis if changed from benign to malignant) was malignant ameloblastoma (40.0%), ameloblastic carcinoma (29.1%), clear cell odontogenic carcinoma (12.7%), primary intraosseous squamous cell carcinoma and ameloblastic fibrosarcoma (both 5.5%). In total, there were 23 sites for MOT metastasis (Figure 6).

### Recurrence

Across all MOTs, 26.8% of patients developed recurrence. Subgroup analysis of this cohort identified the average patient age at 45.2 years (range 5–89 years) with 67.6% male and 32.4% female. The mandible was the most common site, affecting 77.6% of patients with the maxilla affecting 22.4%.

Expressed as a percentage of all recurrent cases, the most common first histological diagnosis was ameloblastic carcinoma (26.1%), malignant ameloblastoma (19.6%) (benign ameloblastoma was recorded at 10.9%), followed by clear cell odontogenic carcinoma (8.7%), primary intraosseous squamous cell carcinoma (6.5%) and ameloblastic fibroma (5.8%). The most common second histological diagnosis was ameloblastic fibrosarcoma (24.5%), ameloblastic carcinoma (20.4%), malignant ameloblastoma (18.4%) and clear cell odontogenic carcinoma (12.2%).

### Treatment

The most common treatment undertaken was surgical resection of the lesion (87.2%). Some patients also had a neck dissection (25.2%), chemotherapy (9.7%), and radiotherapy (25.6%); these adjuvant treatments were also used however this cohort also included palliative care patients. Enucleation (1.2%) and curettage (3.6%) were also treatments undertaken however for nearly all of such cases, it was not known at that stage if the lesion was malignant or would transform into malignancy.

### Genetics

Fifteen studies undertook some form of genetic analysis on their tumour samples. *BRAF p.V600E* mutations were identified in five of seven tested ameloblastic fibrosarcoma cases. However, immunostaining of the mutant BRAF protein was reported to be unreliable. In the same report, one ameloblastic fibrosarcoma showed a *NRAS p.G61K* mutation which was mutually exclusive with BRAF [16]. This was supported by another case study of a patient with *BRAF p.V600E* mutation in the initial ameloblastic fibroma and also in the subsequently transformed ameloblastic fibrosarcoma [17]. In clear cell odontogenic carcinoma (CCOC), EWSR1 and ATF1 probes have been used to successfully identify gene rearrangements [18–20]. In contrast, sclerosing odontogenic carcinoma was shown to be consistently negative for EWSR1 rearrangements [21–24]. Regarding numerical genetic abnormalities, one case of CCOC had a DNA-Index of 1.93% and an S-Phase of 10.2% [25]. Moreover, in CCOC another article reported gains in chromosomes 14, 19 and 20; and losses in chromosome 9, whereas in the pulmonary metastatic lesions the losses were in chromosome 6 [26].

Sequencing with a mutational panel of two odontogenic carcinomas with dentinoid demonstrated 19 variants in 13 genes. The variants with reported pathological significance were a non-sense variant of APC and missense variant of CTNNB [27]. A CTNNB1 mutation was also identified in a case of ghost cell odontogenic carcinoma [28].

Whole-exome sequencing for a metastasising ameloblastoma demonstrated 7 somatic mutations which included BRAF, MYCN, MLL2, ARIDIA, MLL2, RUNX1, and ASXL1 and no germline mutations. Microsatellite status was also reported as stable with low tumour mutation load (7.0 mutations/Mb) [29]. In ameloblastic carcinoma, aberrant CpG island methylation of p16 was identified however there were no mutational alterations in p53 exons 5–8 [30].

Genetic sequencing did not identify a Patched 1 mutation in a patient with PIOSCC as part of their investigation into basal cell nevus syndrome [31] which has been reported in this review for completeness.

## Discussion

MOTs are a heterogeneous group of lesions that arise from the epithelial and/or mesenchymal remnants of the tooth germ or from a pre-existing odontogenic lesion such as a benign OT or an odontogenic cyst [11]. Due to their low incidence, they remain poorly understood entities with respect to their cancer biology, clinical behaviour, management and long-term prognosis [1, 11, 32]. In addition, their histopathological features significantly overlap with their benign counterparts, increasing the difficulty for the pathologist to reach a diagnosis, and with significant potential for misdiagnosis [3, 32]. Regarding the management of MOTs, surgical excision has been reported as the mainstay of treatment, however this review has highlighted variable use of adjuvant chemo/radiotherapy and neck dissections.

This review showed that MOTs are more common in males, have a strong predilection for the mandible and present in patients with an average age of 45.8 years. Similar to our findings, a retrospective analysis of 308 MOTs in the United States reported a median age range at diagnosis of 50–54 years with a slight male predominance [14]. In general terms, these findings are in agreement with the demographic features of benign OTs (excluding hamartomas such as odontoma, or tumours that are more commonly diagnosed during the first two decades of life such as adenomatoid odontogenic tumour, unicystic ameloblastoma and ameloblastic fibroma) [33]. The exception to this trend in the case of MOTs is GCOC, which has a maxilla:mandible ratio of 2:1 [34, 35].

The current 2017 WHO Classification of Head and Neck tumours describes a range of MOTs, namely: ameloblastic carcinoma (AC), primary intraosseous carcinoma, NOS (PIOC), sclerosing odontogenic carcinoma, clear cell odontogenic carcinoma (CCOC), ghost cell odontogenic carcinoma (GCOC), odontogenic carcinosarcoma and odontogenic sarcomas [11]. This list has evolved over the various iterations of the WHO classification.

### Diagnosis, Terminology, and Classification

The diagnosis of malignant odontogenic tumours is challenging and requires clinical, radiological and histological data to make a definitive diagnosis of malignancy. We have found that for many cases in the literature this full set of data is not presented, making detailed assessment of the accuracy of diagnosis very difficult. Furthermore, applying the usual defining criteria for malignancy (invasion, metastasis and other cytological/histological features, which are at times rather vague and exist on a spectrum) in isolation to odontogenic lesions may result in overdiagnosis. There are several reasons for this, pertinent to the particular context in which odontogenic tumours arise. For example, benign tumours, such as ameloblastoma, may widely infiltrate the medullary cavity of the jaw bones and can infiltrate soft tissue. The presence of perineural spread causes diagnostic difficulty as the fact that odontogenic epithelium normally lies in close proximity to nerves must be considered. Furthermore, the presence of cytological atypia in some lesions (more common in the maxilla, largely within the spectrum of benign lesions) raises the issue of which features, and what degree of cytological atypia are associated with malignant behaviour clinically and on imaging. These are important considerations as, in many cases, the diagnostic issues arise in the differentiation from benign tumours. Furthermore, reported deaths from odontogenic tumours do not support a diagnosis of malignancy *per se*, as, for example, inadequate treatment of a maxillary ameloblastoma may result in infiltration of the skull base and infratemporal fossa. Unfortunately, these crucial issues are mentioned only in passing in a small proportion of the literature we have examined.

The evaluation of literature in this systematic review has demonstrated the variable and interchangeable use of the terms malignant ameloblastoma, ameloblastoma with atypia or high-grade features, ameloblastic carcinoma and metastasizing ameloblastoma, which compounds the difficulties in making and reviewing the diagnosis. In the 1971 WHO classification of odontogenic tumours, cysts and allied lesions [36], the term “malignant ameloblastoma” included all neoplasms with ameloblastoma-like features in the primary and metastatic growth, regardless of cytological features of malignancy [36]. Subsequently, in 1974, Shafer and colleagues introduced the term “ameloblastic carcinoma” referring to a well-differentiated ameloblastoma with malignant transformation of the epithelial component. A decade later in 1984, Slootweg and Muller [37] highlighted that the WHO definition of “malignant ameloblastoma” was confusing and proposed that the term “malignant ameloblastoma” should be reserved for lesions that metastasise, despite their well-differentiated appearances; whereas ameloblastic carcinoma should describe lesions that combine features of ameloblastoma with less-differentiated areas (regardless of their histogenesis; encompassing *de novo*, malignant transformation from an ameloblastoma or from an odontogenic cyst) [37]. The WHO 1992 classification continued to use the term “malignant ameloblastoma”; however the definition was clearer in terms of the histologic features, which included any lesion with features of an ameloblastoma with cytological features of malignancy in the primary tumour in the jaws and/or any metastatic growth, regardless of their pathogenesis (primary malignant or malignant transformation of a pre-existing ameloblastoma) [38].

The WHO 2005 classification made significant changes to the classification of malignant odontogenic tumours which were mainly centred on the histogenesis of the lesion [40]. This version included within the odontogenic carcinomas group metastasizing ameloblastoma (a histologically benign ameloblastoma that metastasizes) and defined three subtypes of ameloblastic carcinoma, the first referred to the primary type as corresponded to an ameloblastic carcinoma that arises *de novo* and, two secondary subtypes that arise in a pre-existing ameloblastoma (intra or extraosseous). The term “malignant ameloblastoma” was not used. The current WHO classification (2017) simplified the classification, prioritising the histomorphology above the origin and biological behaviour, leaving out any unproven references to histogenesis or precursor lesions [11]. Ameloblastic carcinoma was included in the odontogenic carcinoma subgroup, referring to the malignant counterpart of an ameloblastoma; whereas metastasizing ameloblastoma was removed from the malignant group and included in the benign epithelial odontogenic tumours group and defined as an ameloblastoma that metastasizes despite its benign histological features [11], making the terminology consistent with metastasising pleomorphic adenoma and other benign conditions with distant spread. However, Reichart et al. [41] have suggested that the current classification has been oversimplified, as they see no clear justification for classifying metastasizing ameloblastoma within the benign group, as it has a 70% 5-year survival.

The SEER database allows the analysis of large cohorts of data contributing to the understanding of these rare malignancies, nonetheless, one of its limitations is the lack of a centralised pathology review and therefore, there is a possibility of ambiguous sub-classification and misdiagnosis [4, 15]. As mentioned before, the SEER database (in common with many others) follows ICD-10 coding of tumours. The ICD-10 classification does not map well onto the current WHO classification of tumours, and still utilises the general term malignant odontogenic tumour (9270/3) as one of the main diagnostic codes. Currently, this includes ameloblastic carcinoma, PIOSCC and sclerosing odontogenic carcinoma. The code for malignant ameloblastoma has been retained (9310/1) and is presented as a synonym for metastasising ameloblastoma. Whilst the individual diagnostic categories do appear to be accessible in recent SEER data, this level of granularity was not available in the 1973–2011 data reported by Lee et al. [4] and Agarwal et al. [14]. This poses a difficulty as it is likely that the categories reported in these large series are almost certainly a mixture of a number of different entities, with varied clinical courses and outcomes. In particular, the continued use of the term “malignant ameloblastoma,” which was defined in the 1972 and 1992 WHO classifications, but which now is depreciated terminology, has resulted in confusion in the literature, as evidenced by the large number of cases of “ameloblastoma with atypia and/or metastases” for which this term has been used, in preference to ameloblastic carcinoma. Given this, the group probably contains a mixture of benign (Am) and malignant (AC) lesions with no certainty as to the proportion which are truly malignant. This has a direct impact on the prevalence of “malignant ameloblastoma,” which has been estimated to be 60% of MOTs in two different reports, although a high percentage of these cases did not metastasise [4, 14].

In order to achieve consistency, the following classification of epithelial odontogenic tumours containing epithelium with the histological pattern of ameloblastoma (at least focally), which is essentially a restatement of the 2017 WHO classification, should be used:

1. Ameloblastomas are considered benign if they have no clinical, radiological or histological features suggestive of malignancy, including no evidence of cytological atypia, malignant transformation or metastasis.
2. Cytologically and otherwise clinically/radiographically benign ameloblastomas which metastasise. The benign appearance must be present in both the primary tumour and the metastasis. The survival data presented in the case series by Slootweg and Muller [37] has been used to suggest that this should be considered as a distinct entity and not classified with benign ameloblastoma, but there is insufficient evidence to define this as a malignant neoplasm. If future molecular studies indicate that metastasising ameloblastoma has similar genetic aberrations to non-metastasising ameloblastoma or to ameloblastic carcinoma, then that may be the time to consider a change in the classification. At present this should be separately classified as metastasising ameloblastoma.
3. Tumours with clinical, radiological and histological features of malignancy and histological pattern of ameloblastoma should be termed ameloblastic carcinoma, regardless of the presence of metastases. Histological features alone are insufficient for this diagnosis. In some cases, in a pre-existing ameloblastoma, this may be very focal, and whilst the term “*in situ*” ameloblastic carcinoma has been suggested, there is currently no literature to support this concept, or any indication that tumours with only focal malignant change behave differently from a benign lesion. The pathogenesis (i.e., from a benign OT or *de novo*) may be interesting to note, but there is little evidence that this has a bearing on clinical behaviour.

At present, there is limited genetic research into the components of this family of MOTs, although some data is appearing from ameloblastic carcinomas [40, 42]. Further insight into each of the main subgroups as described will help elucidate key biological events that identify tumours at risk of malignant transformation and risk of metastasis and further improvements in prognostication and standardisation in treatment algorithms. A diverse range of names, without clear definitions, is likely to hinder research progress and as demonstrated in this review, complicate consolidation of published evidence.

In the present review, the most common second histologic diagnosis was ameloblastic fibrosarcoma (AFS). Regarding this entity, the classification for odontogenic sarcomas has been simplified from 2005 to 2017, and currently it covers a group of mixed odontogenic tumours in which only the mesenchymal component shows malignant cytological features [11]. The former classification included AFS, ameloblastic fibrodentinosarcoma and ameloblastic fibro-odontosarcoma, and whilst these remain as subtypes, the clinical relevance of this subclassification has been called into question [43]. A recently published series of seven cases of AFS, reported that four of these showed evidence of a pre-existing benign precursor such as ameloblastoma or ameloblastic fibroma [16]. The authors stated that no recurrences, metastases, or deaths were seen with a median follow-up of 37 months, however 2 patients were lost to follow-up post-surgery. However, our review found that 12.5% of AFS cases had an initial histological misdiagnosis, many of which were AF, but this also included ameloblastoma and other unspecified odontogenic tumours. The variety of initial diagnoses, some without an odontogenic mesenchyme component raises the issue of misdiagnosis (see later). In these tumours the stromal component should be carefully assessed in order to differentiate mature fibrous tissue from an immature mesenchyme that resembles dental papilla [44]. Given that a large proportion of the reported cases of AFS occur in young adults and children, this is a major potential pitfall, in which lesions which are not neoplastic may be mistaken by the inexperienced pathologist for benign or even malignant mixed odontogenic tumours. Again, it is important to restate that the clinical, radiological and histological features must all be considered before a diagnosis of AFS is made.

In the 2005 WHO classification, PIOSCC was divided into a solid type, those derived from keratocystic odontogenic tumour and a third group derived from odontogenic cysts, nevertheless in the current classification it is represented by a single entity termed Primary intraosseous carcinoma [PIOC (NOS)]. The removal of subtypes and simplification is likely to represent no change in treatment or clinical outcomes with either subtype [43, 45].

In relation to other odontogenic malignancies, a new entity of Sclerosing Odontogenic Carcinoma was added to the WHO Classification of Head and Neck Tumors (2017). There have been 14 cases published to date [46]. Features such as perineural invasion were cited to support its recognition as a malignant tumour, however it has a low mitotic activity and no metastases have been reported to date [46]. Indeed, the diagnostic criteria for this entity are still somewhat vague.

In addition, the current WHO (2017) classification included odontogenic carcinosarcoma (OCS). This was described in the 1992 classification and then excluded from the 2005 classification [38, 39]. This is an extremely rare and aggressive entity, characterised by epithelial and mesenchymal components with cytologically malignant features, with a high positivity for p53 (90%) and Ki67 proliferation index (45%) in both components [47]. A systematic review showed 9 cases of OCS, from which only 1 in 5 arose *de novo* and the remaining were derived from a pre-existing lesion such as ameloblastoma, ameloblastic fibroma and a cementomatous/osteoblastic lesion [48]. As with other entities within the group of MOTs, the pathogenesis of its malignant transformation remains unclear, however multiple recurrences have been reported [49]. Recurrence of OCS has been highly reported in literature with 50% of the patients having metastasis (lungs, lymph nodes, ribs and pelvis) and 57% of the patients succumbing to this malignancy [48].

### Histological Misdiagnosis

Histological misdiagnosis was either directly reported by the authors of the original case or interpreted by the authors of this review during full text analysis in some cases. The latter included an immediate change in diagnosis on review of the sample, clinical progression/deterioration following a benign diagnosis, with subsequent confirmation of a malignant counterpart in the same site or the authors stating a clear MOT was diagnosed and on review of the initial biopsy, the same features were identified even if more subtle. One study has reported that only nine of 22 reported MOTs were actually considered malignant on review [1], which is concerning, and is somewhat supported by the balance of this literature review. Unfortunately, it is likely that misdiagnosis will be frequent, considering that odontogenic tumours are rare, expertise is limited, and definitions varied.

An important factor to consider during the diagnosis of OTs is the interpretation of incisional biopsies, which may not be representative of the entire lesion. Caution in the diagnosis needs to be communicated to the clinical team if they have provided an incisional biopsy that may not be representative, as, for example, ameloblastomas, even with a benign appearance, form a high proportion of OTs cases that have distant metastasis.

Clinicopathological and radiological correlation is essential in the diagnosis of a MOT, and the use of molecular markers may be helpful in addition to this. Within the clinicopathological features, larger lesions, rapid destructive growth, lymph node metastases and older patients are some of the clinical features that are consistent with malignancy [50]. Correspondingly, benign OTs such as ameloblastoma and calcifying epithelial odontogenic tumour (CEOT) are infiltrative and destructive lesions and may be confused with a MOT based on clinical behaviour. At the microscopic level, cellular crowding and budding, nuclear and cellular pleomorphism, high proliferation rates, focal necrosis and perineural or vascular infiltration represent highly suspicious features of malignancy [1, 44, 50]. Again, there is an overlap with benign OTs: tumours such as CEOT and some maxillary ameloblastomas can show some of these histological features in spite of their benign nature [50].

Ki67 may be useful for the differentiation between ameloblastoma and ameloblastic carcinoma. Hunter and Speight suggested that Ki67 expression in more than 20% of the epithelial cells may be consistent with the diagnosis of ameloblastic carcinoma, but this has to be interpreted in the context of the other clinical, radiological and histological features [44]. Other markers, such as SOX2 and OCT4 expression, have also been suggested [51]. To date there is no convincing evidence that the use of immunostaining surpasses histopathology alone. Instead, morphology, immunostaining and cytogenetic techniques should complement each other for diagnosis, especially when the proliferation rate and the presence of cell variants are ambiguous.

In addition, tumours with clear cells, such as CCOC and clear cell variants of intraosseous mucoepidermoid carcinoma and ameloblastic carcinoma, have been shown to lack features of cytological atypia leading to misdiagnosis [50]. Therefore, caution must be exercised during histopathological assessment especially in tumours with ameloblastoma-like epithelium in terms of their proliferation rate, tumours with mixed components and clear cell tumours. For the latter, PAS+/-Diastase and Alcian blue may be used to rule out mucin or glycogen accumulation; with immunostaining for RCC/CD10 and S100/SOX10 helpful to rule out renal carcinoma and melanoma, respectively [50].

### Treatment

In the literature examined, there is general agreement that MOTs should be treated with surgery but this review highlights variable use of adjuvant therapy and neck dissections. Within the spectrum of MOTs, the range of aggressiveness of the malignant process varies, and this is reflected in the varied treatment approaches. Under ‘surgery' we included not only marginal and segmental resections, but also enucleation and curettage in specific cases. Neck dissection was performed in half of all PIOSCC cases and 30% of CCOC cases. In the case of AFS, only 5.3% of the patients had a neck dissection. The combination of surgery and radiotherapy was performed in 37.9% of PIOSCC and 25.4% of ameloblastic carcinoma cases. In the same way, the combination of surgery and chemotherapy was conducted in 15.8% of AFS cases and 13.8% of PIOSCC cases. A survival study of patients with MOTs showed that patients who were not treated with radiotherapy were almost 20 times more likely to die [14].

The most common site of metastasis reported were the cervical lymph nodes which raises the issue of the need for a concurrent neck dissection during surgical resection. In this review, we found that 9.3% of the patients had positive lymph nodes on first presentation. This is within a similar range to the seventeen percent reported by Agarwal et al. [14]. This review has highlighted that the majority of lymph node metastasis from MOTs occur from PIOSCC, malignant ameloblastoma and clear cell odontogenic carcinoma (accounting for a combined 86.5% of all lymph node metastases). Ameloblastic fibrosarcoma was only involved in 2.7% of all lymph node metastases however made up 21.2% of all second histological diagnoses. Further work is required to produce standardised treatment algorithms including concurrent neck dissections.

The challenge arises given that many MOTs are diagnosed on resection rather than the initial biopsy (Table 2): however, re-operating to achieve optimum surgical margins should include consideration of the need for a neck dissection. There is, however, no data to support this as a routine procedure in a clinically N0 neck, and such a case would benefit from multidisciplinary team input into the decision-making process.

Distant organ metastasis (DM) most commonly affected the lungs (30.6% of DM patients), followed by the brain (7.5%), mediastinum and bone (both 3.8%), liver (3.2%), skull (2.7%), and spine and skin (both 2.2%). Lung involvement was in keeping with what has been previously reported for metastasising ameloblastoma [52], ameloblastic carcinoma [9] and CCOC [10]. A retrospective analysis reported only 4% (13/308) of MOTs with distant metastasis at the time of the first diagnosis [14].

### Recurrence and Survival

We report that 26.8% of patients with MOTs developed recurrence with ameloblastic carcinoma and lesions described as malignant ameloblastoma are the most common recurring tumours. Recurrences have been reported in 28% of ameloblastic carcinomas [53], whereas a recent review of CCOC reported that this tumour has a recurrence rate of 38 of 88 (43%) cases [54]. Huang et al. reported that for PIOSCC, the probability of recurrence is related to lymph node metastasis and treatment undertaken, with a 55.6% recurrence rate at 2 years [55].

In terms of survival rates, based on the information from the SEER database, Agarwal et al. [14] reported that patients diagnosed with malignant ameloblastoma had a better survival rate than patients with other “malignant odontogenic tumours” (which included odontogenic carcinomas, PIOSCC and ameloblastic carcinoma, as noted earlier), or AFS [14]. The latter showed the lowest 5-year survival rate of 44.4% compared to the 86.5% seen in malignant ameloblastoma [14]. Increasing age at diagnosis, lack of radiation treatment after surgery, increasing tumour size, and AFS histopathology were each found to exert a statistically significant negative impact on survival [14]. As stated earlier, it was not possible to make clear conclusions regarding survival from this systematic review.

### Molecular Pathology

The molecular mechanisms underpinning MOT development remain poorly understood, mainly due to the rarity of these lesions and thus the difficulty of collating large cohorts with follow-up that allows robust conclusions in terms of clinical behaviour, outcome, and prognosis. For this reason, to date it has not been possible be establish the significance of the genetic/epigenetic alterations that have been reported in these tumours. Despite this, interesting findings have been reported about the genetic profile of some of these MOTs. Ameloblastic carcinomas, as also seen in their benign counterparts, often harbour the *BRAF p.V600E* mutation, however in a lower frequency that ranges from 23 to 33% [40, 56, 57]. This mutation has been demonstrated in other tumours with ameloblastic morphology such as ameloblastic fibromas and ameloblastic fibro-odontomas [56]. Likewise, it was recently reported that the presence of the same mutation in BRAF in 5 of 7 ameloblastic fibrosarcomas (AFS) [16] and also in a rare and aggressive form of AFS [17]. Moreover, it was shown in one AFS that both the epithelial and mesenchymal components harboured the *BRAF p.V600E* mutation [16].

A case diagnosed as malignant ameloblastoma showed 7 somatic mutations which included BRAF, MYCN, MLL2, ARIDIA, MLL2, RUNX1, and ASXL1. However, no further analysis was undertaken to determine which of these mutations serve as oncogenic drivers or passengers or if these mutations acted variably in timing of effect during tumour development [29]. The patient, who had brain and lung metastasis, received combined chemotherapy including Adriamycin, ifosfamide, and dacarbazine and showed a 90% of reduction in the lung metastases after 6 cycles; however, there was no long-term follow-up [29].

CCOC have a rearrangement of *EWSR1* in more than 80% of cases [11], similar to clear cell carcinoma of the salivary glands. Both entities not only share morphological similarities, but also the EWSR1–ATF1 gene fusion [20]. In other cases, the EWSR1–CREB1 fusion has been demonstrated [58]. Although the identification of EWSR1–ATF1 may be useful for the differential diagnosis between CCOC and other clear cell tumours, any significance for the treatment-decision and prognosis is still unclear.

Regarding GCOC, only 51 cases have been reported to date [35]. Genetic alterations in GCOC have been reported for CTNNB1 p.S33C, CREBBP p.K1741^*^, and MLL2 p.S1997fs^*^4 [59]. Whole-genome sequencing was performed by Bose et al. [60] on a single case, in which copy number gains were reported at SHH, GLI1, JAG1, DTX3, and HEY1, whereas gene fusions were seen at TCF4 and PTPRG. On the other hand, an article which included 2 GCOCs among other OTs, reported that *BRAF p.V600E* was not present in these two cases [61].

Furthermore, MOTs with putative driver mutations, such as BRAF for tumours with ameloblastoma-like epithelium and EWSR1 for tumours highly suspicious of being CCOC, should be considered candidates for mutation assessment. In recent years, a few cases of targeted treatment using BRAF-inhibitor monotherapy (vemurafenib) or combination of BRAF-MEK inhibitors (dabrafenib and trametinib) for ameloblastoma (mainly recurrent or metastasising) have been published [62–66]. These reports showed important size reduction of the tumours and no recurrence during their follow-up (around 30–56 weeks). A common finding among these case reports is the apparent lack of resistance of the tumour towards the therapy (compared to what has been seen in melanoma).

## Conclusion

MOTs are a rare, complex and heterogeneous group of tumours with evolving insight into their genetic profiles and classification. There is significant overlap, both histologically and molecularly with normal tooth development and benign odontogenic tumours [67], thus it has been a challenge for pathologists to establish criteria for diagnosis, with appropriate consistent terminology and classification. As aforementioned, their pathogenesis remains unclear, especially in cases where malignant transformation in a benign lesion has apparently taken place. This review has consolidated the extensive case report-dominated literature and highlighted areas for future research, but the main areas of challenge remain: application of a clear definition of malignancy and the consistent use of terminology. This would be aided by the diagnosis and/or review of all odontogenic tumours within specialist centres. Furthermore, it would be a huge challenge, but with a great impact in the improvement of the biological data within SEER and other databases (such as the DOSAK tumour register), to undertake a retrospective pathology review of the tumours following clarification of terminology and improvement/rationalisation of ICD coding. This will be aided by establishment of an expert consortium to map crucial clinical, radiological and histological features to the molecular changes using “omics” techniques which will help define benign and malignant OTs. A deeper understanding of the molecular background of odontogenic lesions will allow the establishment of a new classification system that brings together the genotypic and phenotypic features of these lesions, in order to improve nomenclature that fits both histology and clinical behaviour, opens avenues for targeted therapy and better prognostication.

## Data Availability Statement

The original contributions presented in the study are included in the article/Supplementary Material, further inquiries can be directed to the corresponding author.

## Author Contributions

KH, CM, and MD developed the concept of the paper and the parameters of the review, reviewed the outcomes of the literature review, and wrote the paper. CM and MD gained PROSPERO registration, designed the data collection tools, collated the literature, and undertook data extraction and initial analysis. All authors contributed to the article and approved the submitted version.

## Conflict of Interest

The authors declare that the research was conducted in the absence of any commercial or financial relationships that could be construed as a potential conflict of interest.

## Publisher's Note

All claims expressed in this article are solely those of the authors and do not necessarily represent those of their affiliated organizations, or those of the publisher, the editors and the reviewers. Any product that may be evaluated in this article, or claim that may be made by its manufacturer, is not guaranteed or endorsed by the publisher.
